# Clinicopathological features and pathogenesis of hepatoid adenocarcinoma of the uterine cervix: a case report

**DOI:** 10.3389/fonc.2025.1551657

**Published:** 2025-07-16

**Authors:** Yuanyuan Liu, Renfeng Zhao

**Affiliations:** GuangXi Academy of Medical Sciences, The People’s Hospital of GuangXi Zhuang Autonomous Region, Nanning, Guangxi, China

**Keywords:** hepatoid adenocarcinoma, alpha-fetoprotein, cervix, clinicopathology, case report

## Abstract

Hepatoid adenocarcinoma (HAC) is a specific extrahepatic adenocarcinoma with hepatocellular differentiation, often associated with elevated serum alpha-fetoprotein (AFP). HAC has been reported to occur in the stomach, ovaries, pancreas, uterus, and other sites. Despite the poor prognosis and few effective treatment options, timely and accurate histopathological diagnosis is essential to optimize clinical management for long-term survival. HAC of the uterine cervix is very rare and occurs mainly in postmenopausal women, and the first symptom is irregular vaginal bleeding in all cases, with elevated serum AFP values. HAC of the cervix is more prone to metastasis than ordinary cervical adenocarcinoma, and its prognosis is worse. The treatment plan still refers to the surgical treatment plan for cervical cancer, and radiotherapy or platinum-based combination chemotherapy is given after surgery. With the development of molecular pathology and next-generation sequencing technology, the pathogenesis of HAC is mainly related to hepatoid differentiation, gene mutation, and inflammatory stimulation. Only two cases have been reported in the literature, and one of the reasons for the lack of reports is the lack of knowledge of the clinical and pathological features of the disease; thus, improving the clinical understanding of HAC is crucial for better identifying patients with HAC of the cervix.

## 1 Introduction

Hepatoid adenocarcinomas (HACs) are adenocarcinomas occurring specifically in extrahepatic organs or tissues with a hepatocyte-like structure and cytological differentiation. The first case of HAC of the gastrointestinal tract was reported by Bourreille in 1970, which was subsequently referred to as AFP-producing gastric adenocarcinoma (1). The disease was formally named HAC by Dr. Ishimura in 1985 (2). A rare case of synchronous HAC involving both the stomach and duodenum was identified (3). The world’s first case of hepatic adenocarcinoma of the cervix was reported by Kato in 2007 (4), and in 2019, Liu Liwei reported the first case of cervical HAC in China. Despite the different sites of origin, all of them have histomorphologic features of adenocarcinoma and hepatocellular differentiation, often accompanied by large amounts of alpha-fetoprotein (AFP) secretion. In this paper, we report the clinical data of a case of cervical HAC.

## 2 Case presentation

**Figure 1:**
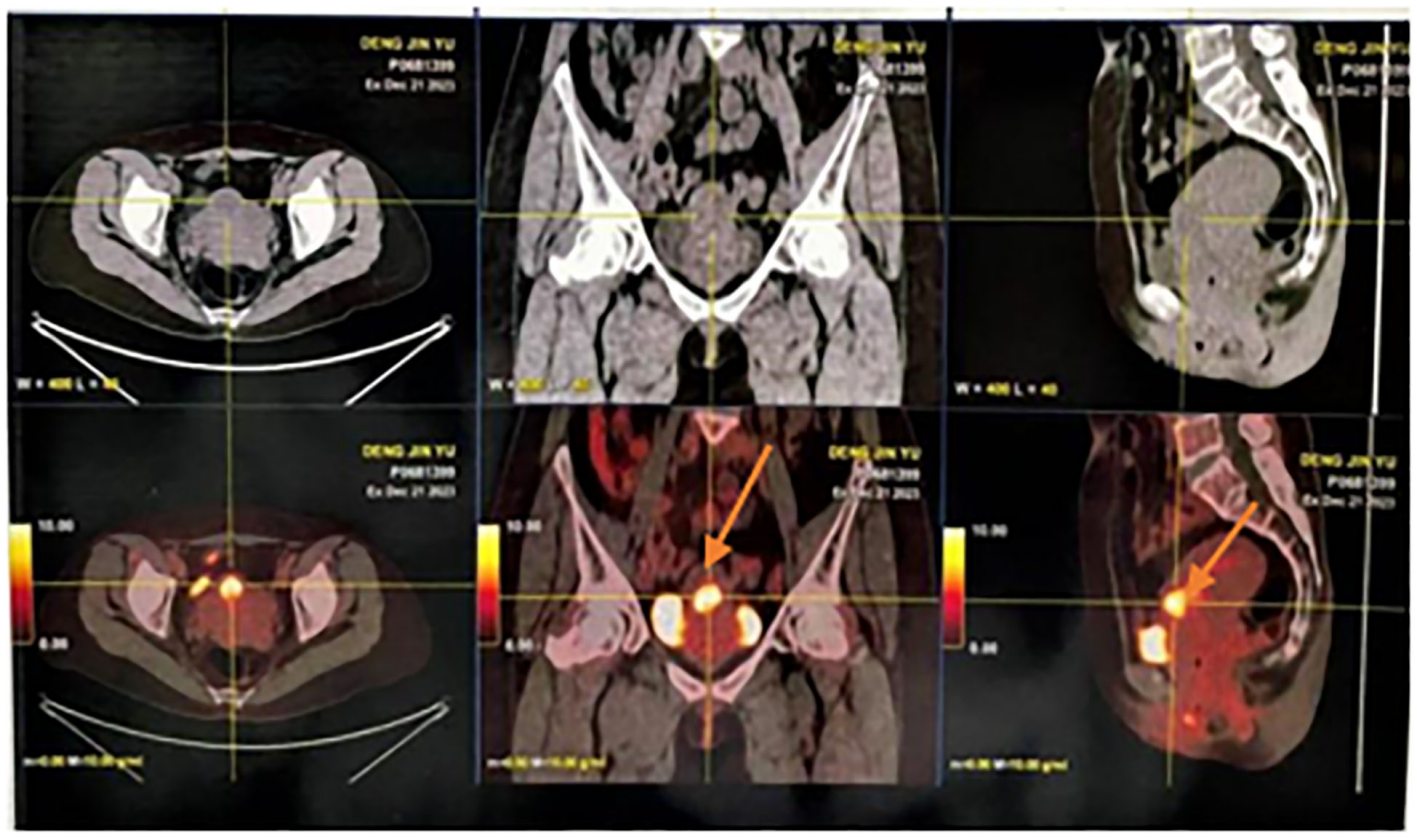
Whole body PET/CT image.

Here, we reported the case of a 51-year-old female patient admitted to the hospital 8 months after symptom onset with persistent elevation of AFP for 8 months and irregular vaginal bleeding for more than 2 months. In April 2023, physical examination found that AFP was 20 ng/mL, and without treatment, irregular vaginal bleeding began in October, with occasional odorous, yellow-colored vaginal fluid. In December, AFP level (10,599.8 ng/mL) was reexamined, and gastroenteroscopy was performed in the Gastroenterology Department, which showed (1) multiple polyps in the stomach; (2) chronic non-atrophic gastritis to be pathologized; and (3) multiple polyps in the colorectum, which were simultaneously treated with high-frequency argon gas knife during the operation (APC procedure). Whole-body PET/CT on 22 December 2023 suggested the following (**Figure 1**): (1) Endometrial thickening of the lower uterine segment and invasion of the anterior wall of the cervix, forming a nodular slightly low-density foci, with still clear margins and a diameter of approximately 2.3 cm. Increased glucose metabolism and patchy foci of increased PDG uptake were seen in the uterine cavity, which, combined with the clinical and pathological examination, suggested the possibility of endometrial hepatic-like adenocarcinoma. (2) Left breast cancer after treatment, with no signs of tumor residue or recurrence in the operation area. The patient underwent surgery for left breast cancer at an outside hospital in 2014 and was on tamoxifen postoperatively until 2022, was switched to anastrozole tablets in 2023, and discontinued the medication in December 2023 on her own.

Endometrial thickening of the lower uterine segment and invasion of the anterior wall of the cervix, forming a nodular slightly hypodense foci with clear margins, approximately 2.3 cm in diameter. Glycemic metabolism was elevated, and patchy foci of increased PDG uptake were seen in the uterine cavity.

**Figure 2:**
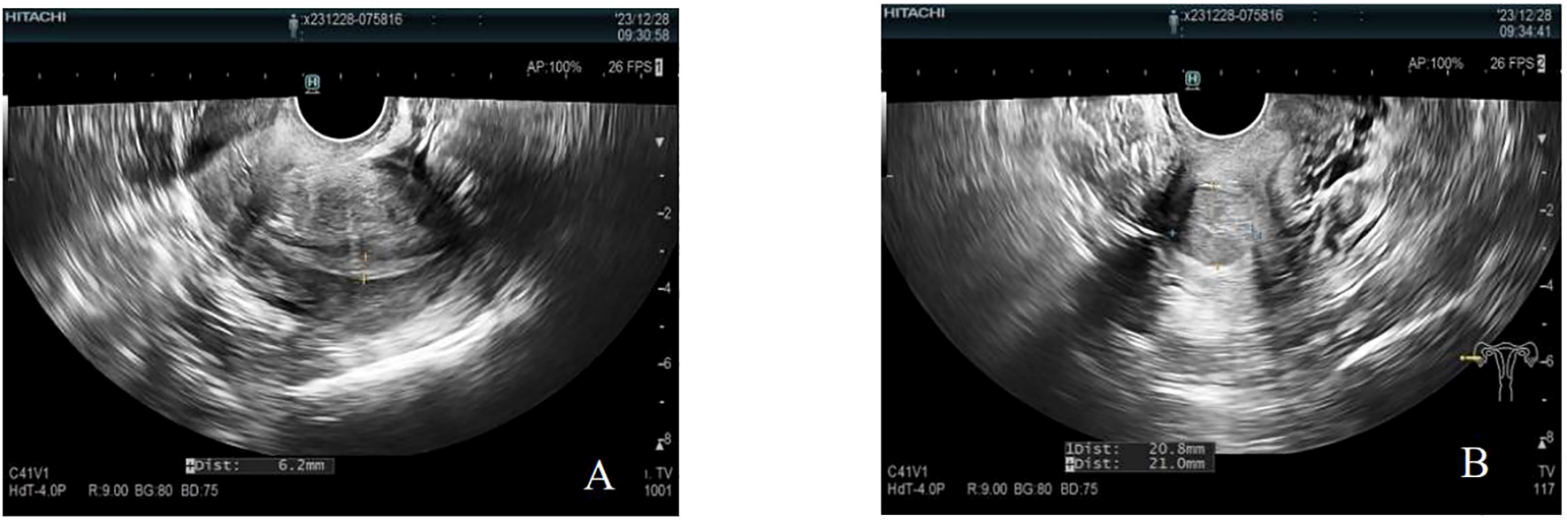
Negative uterus plus double adnexa ultrasound image. **(A)** The endometrium was clear, centered, 6 mm thick, with uneven echogenicity. **(B)** A hypoechoic area was seen in the cervix, approximately 21 × 21 mm in size, with poorly defined borders and poorly homogenized internal echogenicity. CDFI showed no obvious blood flow signal was seen in and around the hypoechoic area of the cervix.

The patient was transferred to our hospital 8 months after symptom onset for further treatment. After admission, AFP measurement was performed: AFP was greater than 3,000.00 μg/L. Carcinoembryonic antigen measurement did not show any abnormality. Transvaginal uterine and double adnexa color Doppler ultrasound revealed that the uterine body is normal. The endometrium was clear, centered, and 6 mm thick with uneven echogenicity. A hypoechoic area was seen in the cervix, measuring approximately 21 × 21 mm, with poorly defined borders and uneven internal echogenicity. Bilateral ovaries were not clear, and no obvious mass echo was seen in both adnexal areas. CDFI showed that no obvious blood flow signal was seen in the hypoechoic area of the cervix and adnexal areas. Endometrial echogenicity is uneven. A hypoechoic area was seen in the cervix. No obvious masses were seen in the bilateral adnexal areas (see **Figures 2A, B**).

The pathology of our consultation suggested a malignant tumor with a tendency towards hepatoid yolk sac tumor, which was difficult to distinguish from HAC. Complete resection of the tumor was recommended for further diagnosis. Immunohistochemistry in our hospital showed the following: Atypical cells AFP(+), Oct3/4(−), Ki-67 (hot spot positive rate approximately 60%), HCG(−), PR(−, positive rate 0%), ER(−, positive rate 0%). Immunohistochemistry: CK(+), SALL4(+), Glypican-3(+), CK5/6(−), P63(−), PAX-8(−), GATA-3(−), SOX10(−), and Vim(−) (for specific significance, refer to **Supplementary Material S1** and **Supplementary Figure S1**). Specialized examination: normal vulva, patent vagina, slight yellow secretion with a foul odor, smooth and enlarged cervix, large uterus, mobility, no pressure, and no palpable mass in both adnexa. Preliminary diagnosis: (1) malignant tumor of the uterus (HAC), (2) postoperative left breast cancer, (3) left adnexal cyst, (4) thyroid nodule, and (5) scarred uterus.

**Figure 3:**
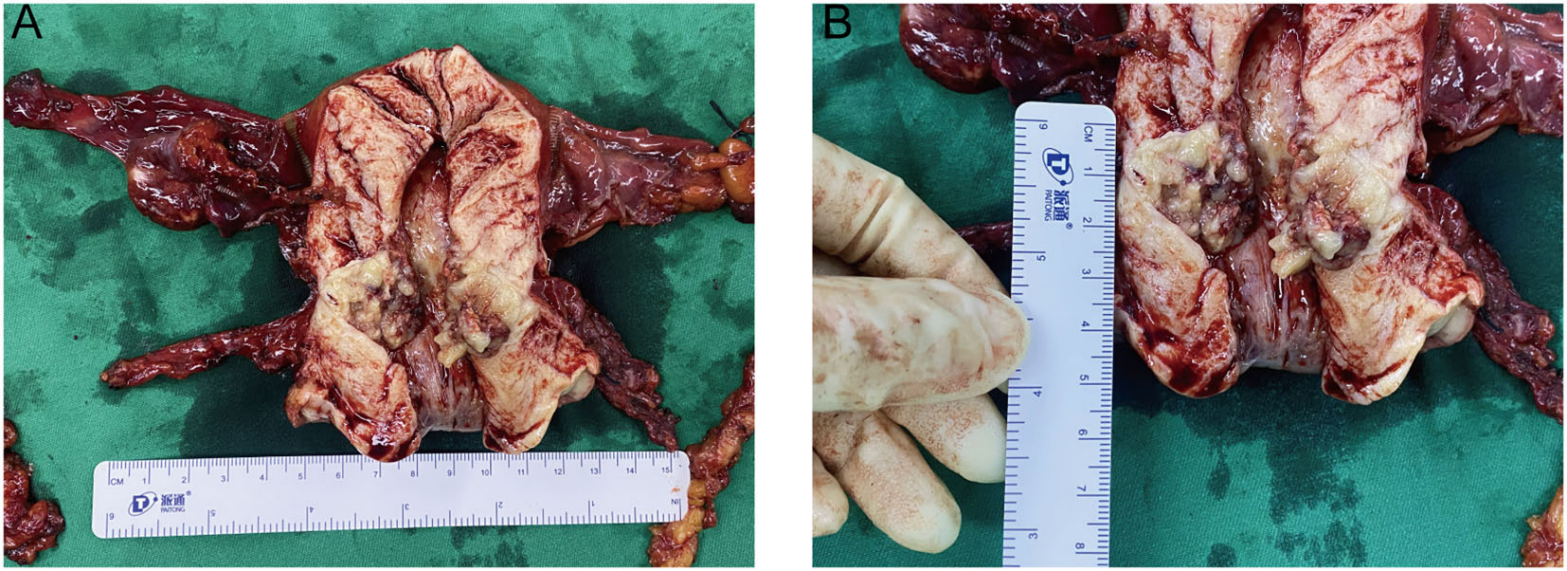
Gross view specimen of the removed uterus and double adnexa. **(A)** Gross specimen of total uterus and double adnexa surgery, uterus size: 11 cm × 10.5 cm × 4 cm. In the lower part of the uterus, a mass was seen, with a size of 2.6 cm × 2 cm × 2 cm; the section was grayish yellow, solid, soft, and friable. Visible tumor foci were located in the endocervical osseous region, endometrium was thin, and there were no obvious foci in the horns of the uterus at the two sides. A vesicle with a thin wall was seen in the left fallopian tube. A vesicle with a maximum diameter of 0.6 cm was seen in the right fallopian tube longitudinal lining with a thin wall. The appearance of both ovaries was not abnormal. **(B)** The ectocervix is smooth and the lesion is located at the endocervix, measuring 2.6 cm × 2 cm × 2 cm; the section is grayish yellow, solid, soft, and friable.

Six months after symptom onset, she underwent transabdominal wide total hysterectomy, bilateral adnexectomy, pelvic lymph node dissection, para-aortic abdominal lymph node dissection, and intestinal adhesion release. During the operation, a nodule was seen in the inter-myometrial wall with a maximum diameter of 0.6 cm, medium texture in the cut surface, and clear peripheral delineation. A grayish yellow, solid mass was seen in the lower uterine segment, with a soft texture in the cut surface. The incision of the left fallopian tube lumen and umbrella is visible, and a vesicle was visible in the tethered membrane, with a maximum diameter of 0.4 cm; the vesicle wall was thin. A vesicle with a maximum diameter of 0.6 cm and a thin wall was found in the mesangium of the right tubal incision (**Figures 3A, B**). Left pelvic lymph node: two pieces of grayish yellow and grayish red tissue, measuring 5 cm × 1.8 cm × 1 cm, and eight pieces of lymph node-like tissue were found, with a maximum diameter of 0.5–2.3 cm. Right pelvic lymph node: a pile of grayish yellow and grayish red crushed tissue, measuring 5 cm × 4 cm × 1 cm, and 19 pieces of lymph node-like tissue were found, with a maximum diameter of 0.2–1.5 cm. Left para-aortic lymph node: a pile of grayish yellow and grayish red crushed tissue, total 2 cm × 1 cm × 0.5 cm, and two lymph node-like tissues were found, with a maximum diameter of 0.9–1 cm. Right para-aortic lymph node: a pile of grayish yellow and grayish red crushed tissue, total 2.5 cm × 1.5 cm × 1 cm, and three lymph node-like tissues were found, with a maximum diameter of 0.5–1 cm.

**Figure 4:**
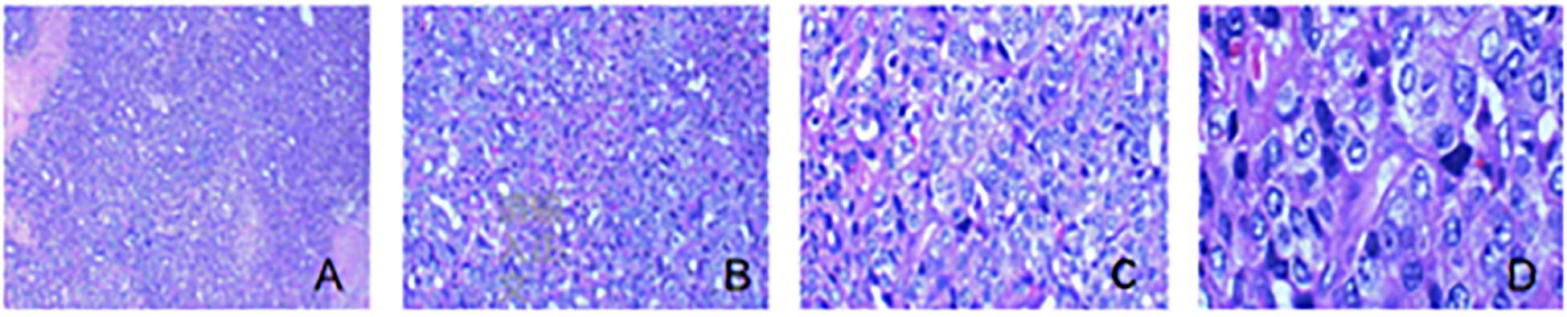
Pathological section of the tumor. **(A)** The tumor showed a diffuse sheet-like and microcystic structure, and focal necrosis was seen (HE, ×40). **(B)** The tumor showed a microcystic structure, and more red transparent spheres were seen forming in the interstitium (HE, ×100). **(C)** The tumor cell cytosol was large; the nucleus was vacuolated, most of which were seen with an obvious nucleolus; and the cytoplasm was rich and eosinophilic, which resembled hepatocyte-like cells (HE, ×200). **(D)** The tumor cell was prone to nuclear schizophrenic image (HE, ×400).

The surgical specimen was sent for pathological examination, under a light microscope. Cervical body junction was seen to be heavily proliferated with atypical cells, arranged in solid sheets and microcysts; tumor cells were polygonal, with vacuolated nuclei and pronounced kernels; nuclear schizophrenia and necrosis were seen. Left pelvic lymph node: Lymph node organization with atypical cells was seen within, and extensive intravascular cancer thrombus was seen. Right pelvic lymph node: Lymph node tissue with atypical cells. Left para-abdominal aortic lymph node: Lymph node tissue, with no atypical epithelioid cell proliferation, was seen. Right para-abdominal aortic lymph node: Lymph node tissue, with no atypical epithelioid cell proliferation, was seen (**Figures 4A–D**).

Postoperative pathological section diagnosis: A large number of atypical cells were seen proliferating at the junction of the cervical body, and the tumor cells were polygonal with vacuolated nuclei, obvious nucleoli, multiple nuclear schizonts, and visible necrosis. Pathological diagnosis was malignant tumor of the cervical canal, inclined to HAC; the maximum diameter of the tumor was approximately 2.6 cm, infiltrated to the deep layer of the cervical canal wall (approximately 1/2 of the cervical canal wall), and upwardly involved the tissue of the endometrial mucous membrane layer; no obvious intravascular cancerous embolus and neural infiltration were seen. Interstitial smooth muscle tumor of the myometrial wall; chronic inflammation of the cervical mucosa; proliferative stage imaging endometrium; chronic inflammation of the mucosa of the right and left fallopian tubes with bilateral median paravalvular tubular cysts; and bilateral ovarian cerebral white matter dystrophy. Metastases were seen in lymph nodes and lymph nodes tissue; no metastasis of cancer was seen. Immunohistochemical findings: hepatocytes, CK(+), Glypican-3(+), CD30(−), SALL-4(+), CK7(+), Ki-67(+), PR(−), ER(−), HCG(−), Oct3/4(−), and AFP(+).

After pathological diagnosis, the patient’s final postoperative diagnosis was as follows: (1) HAC of the uterus, (2) interstitial smooth muscle tumor of the uterine muscle wall, (3) bilateral chronic inflammation of the tubal mucosa, (4) bilateral tubal tethered cysts, (5) postoperative breast cancer on the left side of the body, (6) adnexal cysts on the left side of the body, (7) thyroid nodule, and (8) scarred uterus.

AFP was 2,525.93 μg/L on the 7th postoperative day and 957 μg/L on the 16th postoperative day. The patient was given BEP regimen chemotherapy starting on the 18th postoperative day, every 21 days for a total of four cycles. Bleomycin was no longer used after four cycles of chemotherapy, and consolidation chemotherapy was given for two more cycles after normalization of tumor markers. After completing three cycles of chemotherapy, the patient’s AFP normalized. The patient’s current survival is good, and no tumor recurrence or metastasis has been observed.

## 3 Discussion

In this paper, we report the clinical data of a case of cervical HAC metastasizing in the uterine cavity and analyze the clinicopathological features and pathogenesis of cervical HAC, discussing the four aspects of the clinicopathological features of the diagnosis, differential diagnosis, prognosis, and treatment, and also summarizing the pathogenesis, particularly the role of AFP in tumorigenesis, progression, and metastasis.

### 3.1 Clinical features of HAC

HAC rarely occurs in the uterus; its incidence is less than 0.5%, making it a rare disease (5). Given its rarity, HAC can be misdiagnosed as a yolk sac tumor, as occurred in this case. The present reported case of HAC and the two cases reported by Kato and Liu Liwei, a total of three patients, revealed that cervical HACs occurred mainly in postmenopausal women, with an age of onset of 50–65 years old, and that the first symptom was irregular vaginal bleeding; the serum AFP values were elevated in all of them.

HAC does not exhibit any specific imaging features, but elevated serum AFP is more common in HAC than in common adenocarcinomas, which may aid in early detection. In the 1980s, with the wide application of radioimmunoassay (6), it was found that serum AFP was significantly elevated in some patients with malignant tumors other than hepatocellular carcinoma and yolk cystic tumor, such as HAC. The increase of AFP in the serum of these patients with cancer is the result of synthesis and secretion by cancer cells; thus, AFP is an important tumor marker for HAC. However, approximately 20%–30% of HACs do not produce AFP serum and have normal AFP, but can still be diagnosed as AFP-like adenocarcinoma and called AFP-negative HACs if other major conditions are met.

In this case, the patient was a menopausal woman with elevated serum AFP, and the diagnosis of HAC of the uterine cervix was very clear when considered in conjunction with postoperative histopathology and immunohistochemistry.

### 3.2 Differential diagnosis of hepatoid adenocarcinoma of the uterine cervix

HAC of the cervix needs to be differentiated from tumors of the primary cervix and metastatic HAC, including primary poorly differentiated carcinoma of extrahepatic tissues, hepatoid yolk sac tumors, embryonal carcinoma, and metastatic hepatocellular carcinoma. Hypodifferentiated carcinoma of extrahepatic origin is usually arranged and presented in the form of islands or beams in the localized areas of histology, but immunohistochemistry does not express hepatocyte markers and does not show abnormally elevated serum AFP. Glypican hepatoid yolk sac tumor is morphologically and immunohistochemically very similar to HAC, and both can express SALL4, Glypican-3, and AFP, and often do not express somatic cell markers such as CK7 and EMA. However, histologically, hepatoid yolk sac tumors also have classical S-D vesicles and multicystic structures in addition to liver-like areas, and most of the hepatoid yolk sac tumors occur in young patients with a good prognosis and are sensitive to chemotherapy. The patients with embryonal carcinoma were all young patients, and all had significantly elevated blood human chorionic gonadotropin levels; metastatic hepatocellular carcinoma can be very similar in morphology to HAC, but it does not express SALL4, and imaging suggests that there is a primary lesion in the liver.

This case occurred in an elderly female patient with normal HCG; histology did not show typical multicystic structure and S-D vesicles, expressing CK8/18; imaging, pathohistology, and immunohistochemistry suggested that the tumor was primary and there was no space-occupying lesion in the liver, all of which supported the diagnosis of HAC.

### 3.3 Prognosis of hepatoid adenocarcinoma of the uterine cervix

HAC of the cervix is more prone to metastasis and has a worse prognosis than ordinary cervical adenocarcinoma. The most common sites of metastasis are lymph nodes (57.5%) and liver (46.3%), followed by lungs (3.4%) (7, 8). The majority of patients are diagnosed in advanced stages and nearly 2/3 of patients die within 1 year. AFP greater than 500 ng/mL, TNM stage, and liver metastasis were adverse factors affecting the prognosis, while radical surgery significantly prolongs patient survival. In a study that included 51 patients with lung HAC (8, 9), patients who underwent radical surgery had a 2-year survival rate of 62.5%, whereas those who did not undergo radical surgery had a 2-year survival rate of only 12.5%. No cervical adenocarcinoma-related survival data have been reported.

Our patient was diagnosed as having cervical HAC on postoperative pathology. The tumor involved endometrial mucosal layer tissue upward, and metastases were seen in the left pelvic lymph nodes (5/8) and the right pelvic lymph nodes (2/19). Our patient was diagnosed as having cervical HAC at stage IIIC1p, and no distant metastases were detected by imaging examinations. No tumor recurrence was seen in the postoperative follow-up so far for 8 months. Therefore, improving clinical awareness of HAC is crucial for better identification of patients with cervical HAC.

### 3.4 Treatment of hepatoid adenocarcinoma of the uterine cervix

The current treatment approach for cervical HAC remains largely based on the surgical management protocols for conventional cervical adenocarcinoma, typically followed by adjuvant radiotherapy or chemotherapy. However, there is no standardized chemotherapy regimen specifically for HAC. Platinum-based combination therapy (e.g., cisplatin) remains the most commonly used first-line option for both neoadjuvant and palliative chemotherapy. Several small-scale retrospective studies and case reports suggest that HAC may exhibit poor chemosensitivity and a high likelihood of drug resistance, highlighting the need for further evaluation of its response to chemotherapy and exploration of alternative treatment strategies.

In recent years, immunotherapy has emerged as the fourth major anti-cancer treatment modality, demonstrating breakthrough efficacy in recurrent and metastatic cervical cancers. High tumor mutational burden (TMB), microsatellite instability (MSI), and PD-L1 expression are widely recognized as predictive biomarkers for immunotherapy response. However, research indicates that HAC patients generally exhibit lower TMB compared to conventional adenocarcinomas (7). For instance, gastric HAC ranks fifth in TMB among 33 cancer types, with a median TMB of 3.41 muts/Mb (range: 0.22–69.28 muts/Mb; 10.5% with TMB >10 muts/Mb) (10). Similarly, colorectal HAC has also shown relatively high TMB (11). Nevertheless, TMB data for HAC in other anatomical sites remain unreported, suggesting significant heterogeneity among HAC patients.

Additionally, MSI has been observed in some HAC cases, such as gastric and colorectal HAC. Two studies reported PD-L1 expression rates of 47.6% (10/21) and 59.5% (25/42) in HAC, significantly higher than in adjacent tissues (4.76%) (11, 12). Based on these findings, some experts propose that HAC patients with MSI, PD-L1 positivity, or high TMB may benefit from immune checkpoint inhibitor therapy (13). However, the expression of other immune checkpoints (e.g., CTLA-4) has not been reported, and no data exist on immunotherapy biomarkers in uterine HAC.

While immunotherapy holds promise for select HAC patients, further research is needed to clarify predictive biomarkers and optimal treatment strategies, particularly in cervical and uterine HAC.

### 3.5 Pathogenesis of hepatoid adenocarcinoma

Since the studies of HAC are single case reports and small single-center studies, most of these studies only reported the clinicopathological features, little is known about the pathogenesis of HAC, and the pathogenesis of HAC and the mechanism of elevated blood AFP are still unclear. It is now believed that the pathogenesis is mainly due to the following reasons.

#### 3.5.1 Hepatoid differentiation

It has been proposed that HACs may originate from hepatic differentiation of other tissue components and that these cancer cells are also capable of producing AFP and secreting it into the serum (14).

#### 3.5.2 Gene mutations

Genetic sequencing of HAC has shown that TP53 is the most commonly mutated gene in HAC (11) and is the only gene mutated in HAC at different loci (13).

#### 3.5.3 Epigenetic regulation

CCCTC-binding factor-like (CTCFL) is stably associated with AFP production and is hypothesized to be involved in the pathogenesis of HAC (13).

#### 3.5.4 Inflammatory irritation

All esophageal HACs are located in the lower part of the esophagus, near the gastroesophageal junction. Therefore, some scholars have hypothesized that local microenvironmental alterations due to inflammatory stimuli may play an important role in tumorigenesis.

#### 3.5.5 Gene regulation

AT motif-binding factor-1 (ATBF-1), a transcriptional repressor, is a negative regulator of the AFP gene (15), and AFP expression correlates negatively with ATBF1.

#### 3.5.6 Chromatin abnormalities

Copy number variants (CNVs) tend to be more prevalent in HAC than in common adenocarcinomas, and CNVs in CCNE1, VSTM2B, PLEKHF1, and POP4 are only present in HAC samples (16). The prognosis of patients with CCNE1 amplification is poorer than that of patients without amplification, and could be a new diagnostic marker and therapeutic target.

#### 3.5.7 AFP may be associated with the occurrence, development, and metastasis of hepatoid adenocarcinoma

AFP may be a risk predictor for the prognosis of HAC (17). Existing research has shown that AFP inhibited protein tyrosine phosphatase (PTEN) activity (18, 19) and increased the expression of VEGF and VEGFR proteins (20). Existing studies have shown AFP as a regulator of PTEN-dependent immune-metabolic crosstalk in tumors. Targeting AFP could reverse its immunosuppressive effects by reactivating PTEN/p53 pathways and impairing metabolic adaptations critical for tumor immune evasion (21, 22). Since most HAC patients have elevated peripheral blood AFP levels, it is hypothesized that AFP can inhibit immune responses by suppressing the function of immune cells, thus allowing HAC cells to evade the body’s immune surveillance function (23, 24).

Unfortunately, the patients in this case refused to undergo genetic testing for financial reasons; thus, we did not obtain the results of next-generation sequencing analysis in this patient with cervical HAC.

Given the molecular parallels between HAC and AFP-secreting HCC, therapeutic strategies such as AFP-targeted CAR T cells, immune checkpoint inhibitors (for PD-L1+ cases), and TKIs (e.g., sorafenib) warrant clinical investigation. Multicenter collaborations should prioritize biomarker-driven trials to establish evidence-based therapies for this rare malignancy.

## 4 Conclusion

HAC of the uterine cervix is a rare tumor with atypical clinical symptoms and signs, and the diagnosis is based on postoperative pathological and histological findings. Clinically, women with cervical tumors and elevated AFP need to consider the possibility of cervical HAC. Hepatocellular adenocarcinoma of the uterine cervix is very different from typical adenocarcinoma in terms of bioinvasiveness, prognosis, and clinicopathological features, and it is more aggressive than common adenocarcinoma, more likely to be resistant to chemotherapy, and with a poorer prognosis. Therefore, it is important to accurately recognize and diagnose endometrial HAC in clinical practice. Cutting-edge technologies, such as single-cell sequencing, spatial proteomics, and transcriptomics, will expand our understanding of its heterogeneity and pathogenesis. In addition, we hope that by organizing multicenter basic and clinical studies, more oncologists will focus on HAC and provide new diagnostic and therapeutic approaches (25–27).

Given the extreme rarity of cervical HAC, we advocate for international multicenter registries to aggregate cases, integrate molecular profiling, and accelerate evidence-based management—similar to efforts for other rare cancers.

## Data Availability

The original contributions presented in the study are included in the article/**Supplementary Material**. Further inquiries can be directed to the corresponding author.
