# Supplementary material for: Clinicopathological features and pathogenesis of hepatoid adenocarcinoma of the uterine cervix: a case report
Source: Front Oncol. 2025 Jul 16;15:1551657. doi: 10.3389/fonc.2025.1551657 (PMC12307153; doi:10.3389/fonc.2025.1551657)

Supplementary Material s1

**SALL4**, a zinc finger transcription factor belonging to the SALL gene family, plays a critical role in embryonic development, particularly in maintaining the pluripotency of germ cells and stem cells. SALL4 serves as a core marker for diagnosing yolk sac tumors (YST). Its strong positive expression is pivotal in distinguishing YST from other malignancies, such as hepatocellular carcinoma and adenocarcinoma. However, SALL4 must always be used in combination with other markers (e.g., AFP, Glypican-3, PLAP, OCT3/4) to ensure diagnostic accuracy and avoid overinterpretation based on a single marker.

**Glypican-3 (GPC3)**, a heparan sulfate proteoglycan located on the cell membrane surface and a member of the Glypican family, is highly expressed during embryonic development (particularly in the liver and placenta) but shows significantly reduced or absent expression in normal adult tissues. Glypican-3 is a critical biomarker for hepatocellular carcinoma (HCC) and yolk sac tumors (YST). To distinguish between these two malignancies, GPC3 must be combined with other markers such as SALL4 and AFP for accurate differential diagnosis.

**Oct3/4** is a specific marker for germ cell tumors, particularly seminoma and embryonal carcinoma. In liver tumors, Oct3/4 positivity strongly suggests metastatic germ cell tumor, necessitating further confirmation of the primary site through additional markers (such as SALL4 and AFP) and imaging studies.

**PAX8**, a member of the paired box (PAX) gene family, plays a key role in organogenesis during embryonic development, particularly in the formation of the thyroid, kidneys, Müllerian ducts (female reproductive system), and Wolffian ducts (male reproductive system).

In female genital tract tumors, PAX8 is highly expressed:

Ovarian serous carcinoma and endometrioid adenocarcinoma: PAX8 positivity is observed in ~80-90% of cases, supporting a Müllerian origin (helpful in distinguishing from metastatic carcinomas of gastrointestinal or breast origin).

Cervical adenocarcinoma: Some subtypes (e.g., gastric-type adenocarcinoma) may be PAX8-negative, necessitating the use of additional markers (e.g., p16) for accurate classification.

**SOX10** is a member of the SOX transcription factor family and plays a role in neural crest cell differentiation, melanocyte development, and peripheral nervous system formation.

Cutaneous melanoma: SOX10 is positive in >90% of cases, aiding in the distinction between melanoma and carcinoma or sarcoma (e.g., SOX10+/S100+ supports melanoma).

Schwannoma and malignant peripheral nerve sheath tumor (MPNST): SOX10 expression suggests neural crest origin.

Metaplastic carcinoma: SOX10 is often positive, helping differentiate it from squamous cell carcinoma or sarcomatoid carcinoma.

Salivary gland tumors: SOX10 shows high positivity in adenoid cystic carcinoma and pleomorphic adenoma.

Supplementary Material s2

| Immunohistochemical markers | Hepatoid adenocarcinoma | Ovarian cystic Tumor (YST) | Hepatocellular carcinoma (HCC) |
| --- | --- | --- | --- |
| CK7 | Strong positive | Negative | Negative |
| CK19 | Strong positive | Negative | Negative(If positive, it indicates mixed liver cancer） |
| HepPar-1 | Negative | Negative | Strong positive (High specificity) |
| Arginase-1 | Negative / Weak positive | Negative | Strong positive(High specificity) |
| Glypican-3 | Negative / Focal positive | Strong positive | Positive（50%-70%） |
| AFP | Positive | Strong positive | Positive |
| SALL4 | Negative / Partial positive | Strong positive | Negative |
| CDX2 | Positive(Gastrointestinal origin) | Negative | Negative |
| TTF-1 | Negative（Positive indicates metastasis） | Negative | Negative |
| GPC3 | Negative | Positive | Positive |
| β-catenin | Membrane positive | Negative | Nuclear positive |
| EMA | Membrane positive | Negative | Negative |
| PLAP | Negative | Partial positive | Negative |
| OCT3/4 | Negative | Negative(Only positive for embryonic cancer) | Negative |
| Schiller-Duvel（SD）body | Negative | Positive | Negative |

Supplementary Material s3

It is regrettable that, due to financial constraints, the patient in this case declined all genetic testing, and thus we were unable to obtain next-generation sequencing (NGS) analysis results for this case of cervical hepatoid adenocarcinoma. This has imposed certain limitations on the study of cervical hepatoid adenocarcinoma:

Impact on precise diagnosis: Genetic testing could have helped identify specific genetic alterations, such as certain mutations or gene fusions, enabling more accurate tumor classification and diagnosis, as well as clarifying its true origin and biological characteristics.

Obstruction to treatment planning: The results of genetic testing could have provided critical guidance for targeted therapy. If specific mutations had been detected, there might have been targeted drugs available. Instead, physicians had to rely on empirical treatment options such as traditional chemotherapy or radiotherapy, potentially missing more effective personalized treatment approaches.

Inability to accurately assess prognosis: Certain genetic markers are closely associated with tumor prognosis. Detecting specific genetic alterations through testing would have helped physicians evaluate the patient’s risk of disease progression, likelihood of recurrence, etc. The refusal of genetic testing deprived clinicians of crucial molecular biological information, making it difficult to accurately predict disease progression and complicating the development of comprehensive follow-up and rehabilitation plans.

Detrimental to tumor research: Cervical hepatoid adenocarcinoma is a rare malignancy, and genetic testing would have contributed to accumulating case data, deepening the understanding of this disease, and elucidating its pathogenesis and genetic features. This would have supported future research and the formulation of treatment guidelines. The patient’s refusal to undergo testing hinders progress in related research, impeding medical advancements in the exploration and overall improvement of treatment strategies for this rare disease.

Supplementary Figure s1
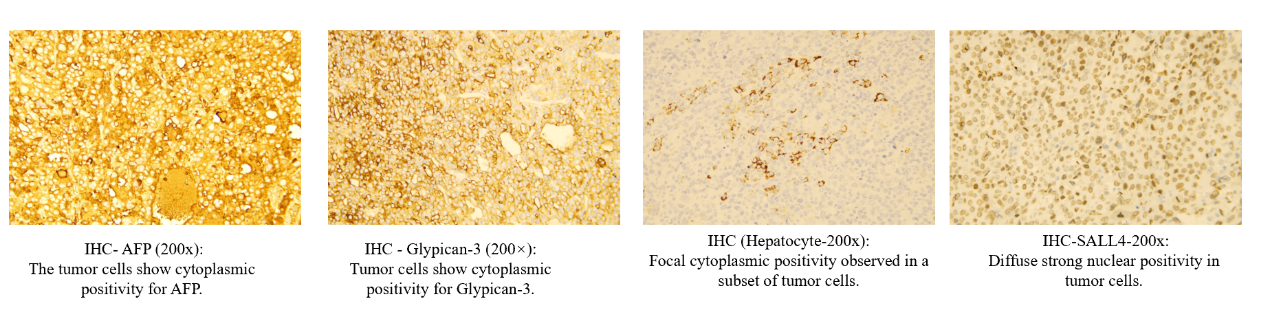

Supplement: Supplementary file 1 [file DataSheet1.docx]
